# Prolonged Maternal Separation Induces the Depression-Like Behavior Susceptibility to Chronic Unpredictable Mild Stress Exposure in Mice

**DOI:** 10.1155/2021/6681397

**Published:** 2021-07-29

**Authors:** Yaoyao Bian, Yanting Ma, Qian Ma, Lili Yang, Qinmei Zhu, Wenlin Li, Lingdong Meng

**Affiliations:** ^1^College of Acupuncture and Massage, College of Regimen and Rehabilitation, Nanjing University of Chinese Medicine, Nanjing 210023, China; ^2^Jiangsu Provincial Engineering Research Center of TCM External Medication Development and Application, Nanjing University of Chinese Medicine, Nanjing 210023, China; ^3^TCM Nursing Intervention Laboratory of Chronic Diseases, Nanjing University of Chinese Medicine, Nanjing 210023, China; ^4^Department of Nursing, Suzhou TCM Hospital Affiliated to Nanjing University of Chinese Medicine, Suzhou 215009, China; ^5^School of First Clinical Medicine, Nanjing University of Chinese Medicine, Nanjing 210023, China; ^6^Jingwen Library, Nanjing University of Chinese Medicine, Nanjing 210023, China; ^7^School of Medicine, Yangzhou Polytechnic College, Yangzhou 225009, China; ^8^Department of Nephrology, Yangzhou Hospital Affiliated to Nanjing University of Chinese Medicine, Yangzhou 225002, China

## Abstract

Early life stress is an important determinant for developing depression later in life. It is reported that maternal separation (MS) could trigger stress sensitivity in adulthood when exposed to stress again. However, it could also result in resilience to stress-induced depression. The conclusions are contradictory. To address this issue, C57BL/6N newborn pups were exposed to either daily short MS (MS for 15 min per day; MS15) or prolonged MS (MS for 180 min per day; MS180) from the first day postpartum (PD1) to PD21. Adult mice were then subjected to chronic unpredictable mild stress (CUMS) exposure from PD64 to PD105. The behavior tests such as the forced swimming test (FST), tail suspension test (TST), and open-field test were performed once a week during this time. Besides, the hippocampal neurosteroids, serum stress hormones, and hippocampal monoamine neurotransmitters were measured at PD106. We found that mice in the MS180 group displayed the reduced struggling time and the increased latency to immobility in both FST and TST. However, there was no significant difference in the MS15 group. The levels of hippocampal neurosteroids (progesterone and allopregnanolone) were decreased, and the serum levels of corticosterone, corticotropin-releasing hormone, and adrenocorticotropic hormone were overexpressed in the MS180 group. Besides, the expressions of monoamine neurotransmitters such as 5-hydroxytryptamine and 5-hydroxy indole acetic acid significantly decreased in the MS180 group, but not in the MS15 group. All findings revealed that prolonged MS, rather than short MS, could increase the susceptibility to depression-like behavior when reexposed to stress in adulthood. However, future studies are warranted to identify the underlying neuromolecular mechanism of the MS experience on the susceptibility to adult stress reexposure.

## 1. Introduction

Depression is a serious mood disorder that affects more than 300 million people globally [1]. It is the 3rd major leading cause of disability-adjusted life-years lost among adolescents [2], and it is predicted to rank the top disease by 2030 [3]. The prevalence of depression is reported as high as 15-18% worldwide [4]. It brings huge social and economic burdens. Considerable evidence indicated that the onset of depression is highly correlated with early life stress (ELS) exposure in childhood [5, 6]. Children who experience ELS are twice more likely to develop depression later in life than those who are not exposed to early adversities [7]. Meanwhile, ELS can alter brain structure and function, exert long-lasting epigenetic impacts on the brain, and make individuals vulnerable to develop depression-like behavior [8]. Thus, early life experiences are important determinants in the development of depression-like behavior in adulthood.

The specific neurobiological mechanism of ELS inducing the susceptibility to depression-like behavior is complex. The longstanding recognition showed that the hypothalamic-pituitary-adrenal (HPA) axis displays a critical mediator in stress response. The adversity stress can affect the secretion of various stress hormones, i.e., corticosterone (Cort), corticotropin-releasing hormone (CRH), and adrenocorticotropic hormone (ACTH), thereby resulting in the negative feedback of HPA axis regulation [9, 10]. Meanwhile, the impaired HPA axis can affect the synthesis of monoamine neurotransmitters, i.e., 5-hydroxytryptamine (5-HT) and 5-hydroxy indole acetic acid (5-HIAA), ultimately leading to depression [10, 11]. Besides, neurosteroid (i.e., progesterone and allopregnanolone) biosynthesis plays an important role in the neurodevelopment of the stress adversities on the resultant behavior responses [12]. They can not only impact the HPA axis function but also mediate neuronal excitability [13].

Rodent models of maternal separation (MS) are often used to study stress and behavioral responsiveness to early life adversities [14]. Mounting evidence showed that the underlying mechanism of MS is associated with neuroinflammatory response and oxidative stress [15–17]. It is reported that MS can trigger stress sensitivity in adulthood when exposed to stress again [18]. However, it can also reduce HPA axis reactivity and result in resilience to stress-induced depression [19]. The conclusions are contradictory. It leads us to suspect that different exposure times in early life may have different impacts on the stress responses later in life.

To further explore the critical effects of different degrees of early life adversities on adult behaviors, daily short MS (MS for 15 min per day; MS15) and prolonged MS (MS for 180 min per day; MS180) in early life experience and chronic unpredictable mild stress (CUMS) in later life were employed in our study. We sought to explore the critical effects of different MS exposure times on the development of depression-like behavior susceptibility in adulthood.

## 2. Materials and Methods

### 2.1. Animals

All care and treatment were carried out following the Guidelines of Accommodation and Care for Animals. And all procedures were permitted by the Laboratory Animal Management Committee of Nanjing University of Chinese Medicine.

Eight pregnant C57BL/6N mice (weighing 28 ± 2 g) provided by the Experimental Animal Center of Nanjing University of Chinese Medicine were used in the current study. The mice were housed in standard cages with free access to food and water at a controlled temperature (22 ± 2°C) and a 12 h light/dark cycle (light on at 06:00). All mice were allowed to acclimatize this condition during the week before and after delivery.

The eight mice were randomly divided into 4 groups, that is, control (number of mothers *n* = 2), CUMS (number of mothers *n* = 2), MS180+CUMS (number of mothers *n* = 2), and MS15+CUMS (number of mothers *n* = 2) groups. The day of birth was the first day postpartum (PD1). The pups were weaned at PD21. It is reported that sex differences can increase the risk of depression by regulating sex hormone fluctuations [20]. To avoid the impacts of sex dependence, six male pups in each group (*n* = 6) were selected at PD21 for the following study. If the dam delivered more than six offsprings, the dam with its pups was kept for further study in each group, and the rest were euthanized. If both dams in one group have more than six pups, just one dam and its pups were kept randomly.

### 2.2. MS Intervention

**Figure 1:**
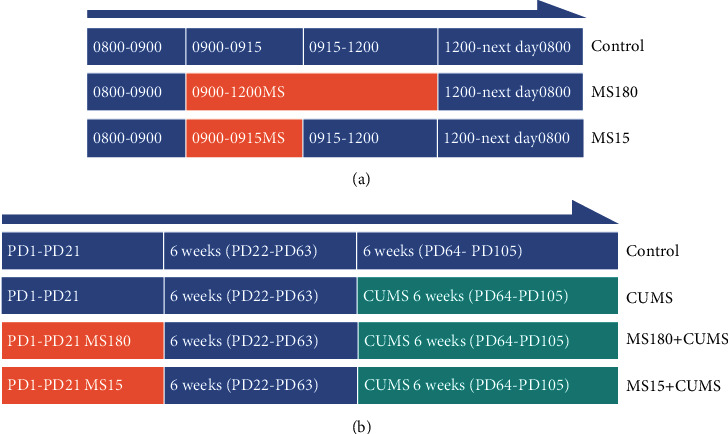
The protocol timeline of MS and CUMS in different groups. (a) Schematic timeline of maternal separation from their dams for 180 min (0900-1200 h; MS180) and 15 min (0900-0915 h; MS15) per day. (b) Schematic timeline of maternal separation and chronic unpredictable mild stress in different groups.

The MS protocol was implemented according to the reported scheme [14], as shown in Figure 1(a). From PD1 to PD21, the pups were separated from their dams and sires (they were moved into a single cage) for 15 min (0900-0915 h, MS15) or 180 min (0900-1200 h, MS180) per day to constitute short or prolonged separation in an incubator (30 ± 2°C). After the daily short or prolonged separation, pups were returned to their home cages and reunited with their mothers. The pups in the controls were handled equally in an incubator (30 ± 2°C) but not separated from their dams and sires.

### 2.3. CUMS Intervention

The CUMS protocol was established by the previous studies [21, 22]. The variate-stressor paradigm was applied in the CUMS, MS180+CUMS, and MS15+CUMS groups from PD64 to PD105. The types and duration of stressors during the one-week intervention were given, as shown in Table 1. The mice were exposed to each stressor separately. The stressors would not appear simultaneously, and the same stressor would not be used for two consecutive days. Mice from PD22 to PD63 in each group were housed without any intervention, and they were free with water and food in the same controlled condition. The CUMS group (*n* = 6) was subjected to CUMS for 6 weeks from PD64 to PD105 without MS. The MS180+CUMS group (*n* = 6) was treated with MS180 from PD1 to PD21 and exposed to CUMS for 6 weeks from PD64 to PD105. The MS15+CUMS group (*n* = 6) was treated with MS15 from PD1 to PD21 and exposed to CUMS for 6 weeks from PD64 to PD105. Controls (*n* = 6) were housed equally without any intervention, as shown in Figure 1(b). The mice were sacrificed to harvest the blood and hippocampus at the end of behavioral tests at PD106.

### 2.4. Behavioral Tests

Behavior assessments including the forced swimming test (FST), tail suspension test (TST), and open-field test (OFT) were performed once a week, that is, PD63, PD70, PD77, PD84, PD91, PD98, and PD105. Smart 3.0 tracking software (Panlab) was applied to record the movements and measure the immobility times of the above behavioral tests. The FST was conducted as a previous study described by Porsolt and Le Pichon [23]. After FST, the mice were allowed to rest for 24 h. The TST was performed as a previous study reported by Steru et al. [24]. After another 24 h rest, the total path of mice in OFT was measured as described previously [25].

### 2.5. Assessment of Neurosteroid Levels

Enzyme-linked immunosorbent assay (ELISA) was used to analyze neurosteroid levels as the previous paper described [26]. Tissues from the hippocampus were collected at the end of behavioral tests on PD106. The tissues were extracted and homogenized using lysis buffer on ice, followed by cold centrifugation at 10, 000 g for 30 min. And then, the supernatants were collected. The levels of progesterone (JEB-15609) and allopregnanolone (JEB-17704) were determined by Enzyme Immunoassay Kits (JinYiBai, China) based on the manufacturer's instruction. Samples (or standard) and conjugate were added to each cell. Then, the plate was incubated for 60 min at room temperature. The wells were washed and incubated with the antibody for another 60 min at room temperature. After washes and color development, the absorbance was measured by an ELISA plate reader (BioTek, USA) at 450 nm.

### 2.6. Assessment of Serum Hormone Levels

The serum hormone levels were analyzed by ELISA too. After the blood was harvested and centrifuged as above, the levels of Cort (JEB-12812), CRH (JEB-12764), and ACTH (JEB-12678) in serum were measured by ELISA kits (JinYiBai, Nanjing, China) according to the instruction. The absorbance was measured as above.

### 2.7. Assessment of Monoamine Neurotransmitter Levels

The levels of monoamine neurotransmitters such as 5-HT (JEB-12689), 5-HIAA (JEB-17702), adrenaline (AD, JEB-12543), norepinephrine (NE, JEB-15641), dopamine (DA, JEB-12552), homovanillic acid (HVA, JEB-17703), and 3,4-dihydroxyphenylacetic acid (DOPAC, JEB-15306) were also analyzed by ELISA kits (JinYiBai, Nanjing, China). Hippocampus were quickly homogenized with lysis buffer on ice and centrifuged at 10, 000 g for 30 min at 4°C. The expressions of the above monoamine neurotransmitters were performed as above.

### 2.8. Statistical Analysis

Results were presented as the mean ± S.E.M. and analyzed using SPSS 17.0 software. Measurements were performed by one-way ANOVA, followed by Tukey's honestly significant difference test and two-factor repeated measures ANOVA in statistical significance analysis. A value of *P* < 0.05 was considered statistically significant.

## 3. Results

### 3.1. Effects of MS Experience on the Depression-Like Behavior Susceptibility to CUMS Exposure in FST

**Figure 2:**
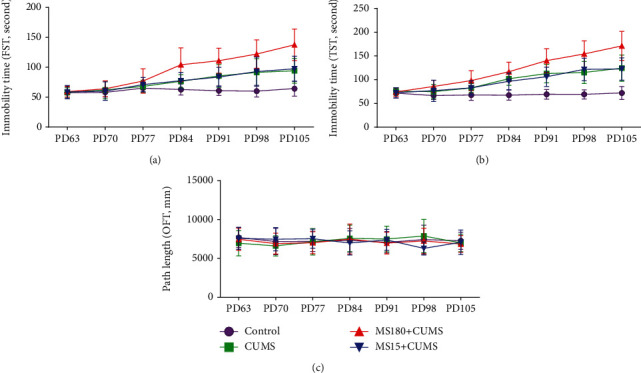
Effects of MS experience on behavioral performance at different time points when exposed to CUMS. (a) The immobility time of different groups at different time points in the FST. (b) The immobility time of different groups at different time points in the TST. (c) The path length of different groups at different time points in the OFT. The data were expressed as the mean ± S.E.M.

In FST, the results of two-factor repeated measures ANOVA showed that the intergroup effect was statistically significant (*F*(3, 20) = 20.363, *P* < 0.001). And the two-two comparison results indicated that the MS180+CUMS group had a markedly increased trend in immobility time at PD84 (*t* = −3.399, *P* = 0.007), PD91 (*t* = −5.487, *P* < 0.001), PD98 (*t* = −5.940, *P* < 0.001), and PD105 (*t* = −6.167, *P* < 0.001) in comparison with the controls. When compared with the CUMS group, there was a significant difference in immobility time in the MS180+CUMS group, as the results of FST suggested PD98 (*t* = −2.281, *P* = 0.046) and PD105 (*t* = −3.188, *P* = 0.010). Besides, the MS180+CUMS group revealed a remarkable increase in immobility time at PD91 (*t* = 2.530, *P* = 0.030) and PD105 (*t* = 2.923, *P* = 0.015) when compared with the MS15+CUMS group. However, there was no statistical significance (*P* > 0.05) between the MS15+CUMS and CUMS groups, as shown in Figure 2(a) and Table 2. All these findings revealed that MS180 could induce the depression-like behavior susceptibility to CUMS exposure in adulthood, as the immobility time in FST was significantly increased.

### 3.2. Effects of MS Experience on the Depression-Like Behavior Susceptibility to CUMS Exposure in TST

In TST, the results of two-factor repeated measures ANOVA indicated that the intergroup effect was statistically significant (*F*(3, 20) = 37.637, *P* < 0.001). And the two-two comparison results show that there was a significant difference in the MS180+CUMS group at PD70 (*t* = −3.073, *P* = 0.012), PD77 (*t* = −3.101, *P* = 0.011), PD84 (*t* = −5.245, *P* < 0.001), PD91 (*t* = −6.388, *P* < 0.001), PD98 (*t* = −7.161, *P* < 0.001), and PD105 (*t* = −7.223, *P* < 0.001) when compared with the controls. In comparison with the CUMS group, the MS180+CUMS group suggested a markedly increased trend of immobility time at PD98 (*t* = −2.672, *P* = 0.023) and PD105 (*t* = −2.777, *P* = 0.020). Moreover, the MS180+CUMS group exhibited a remarkable increase in immobility time at PD91 (*t* = 2.571, *P* = 0.028) and PD105 (*t* = 3.080, *P* = 0.012) when compared with the MS15+CUMS group. However, there was no significant difference (*P* > 0.05) between the MS15+CUMS and CUMS groups, as shown in Figure 2(b) and Table 2. All the results suggested that MS180 could evoke depression-like behavior susceptibility to adult CUMS exposure, as the immobility time in TST was markedly increased.

### 3.3. Effects of MS Experience on the Behavioral Response to CUMS Exposure in OFT

In OFT, the results of two-factor repeated measures ANOVA showed that the intergroup effect was not statistically significant (*F*(3, 20) = 0.071, *P* = 0.975) among the groups at PD63, PD77, PD84, PD91, PD98, and PD105. It indicated that there was no significant difference in locomotor activity in CUMS mice induced by daily short MS or prolonged MS, as shown in Figure 2(c) and Table 2.

### 3.4. Effects of MS Experience on the Levels of Neurosteroids at PD106 When Exposed to CUMS

**Figure 3:**
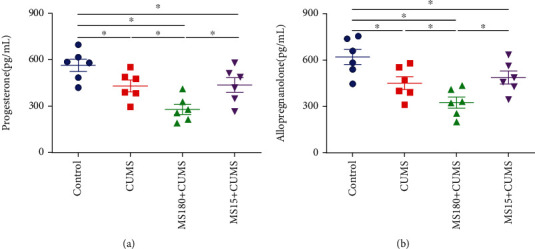
Effects of MS experience on hippocampal neurosteroid levels when exposed to CUMS. (a) The effect of MS on the progesterone levels when exposed to CUMS. (b) The effect of MS on allopregnanolone levels when exposed to CUMS. ^∗^*P* < 0.05 between the two groups.

When compared with the controls, the levels of progesterone and allopregnanolone were lower in the CUMS group. In comparison with the CUMS group, the levels of progesterone (*F* (3, 20) = 8.635, *P* < 0.05; Figure 3(a)) and allopregnanolone (*F* (3, 20) = 8.164, *P* < 0.05; Figure 3(b)) had a significantly decreased trend in the MS180+CUMS group. However, there was no significant difference between the MS15+CUMS and CUMS groups (*P* > 0.05). These results revealed that the dysfunction of progesterone and allopregnanolone levels induced by prolonged MS might contribute to depression-like behavior susceptibility to CUMS exposure in adulthood.

### 3.5. Effects of MS Experience on the Levels of Serum Hormones at PD106 When Exposed to CUMS

**Figure 4:**
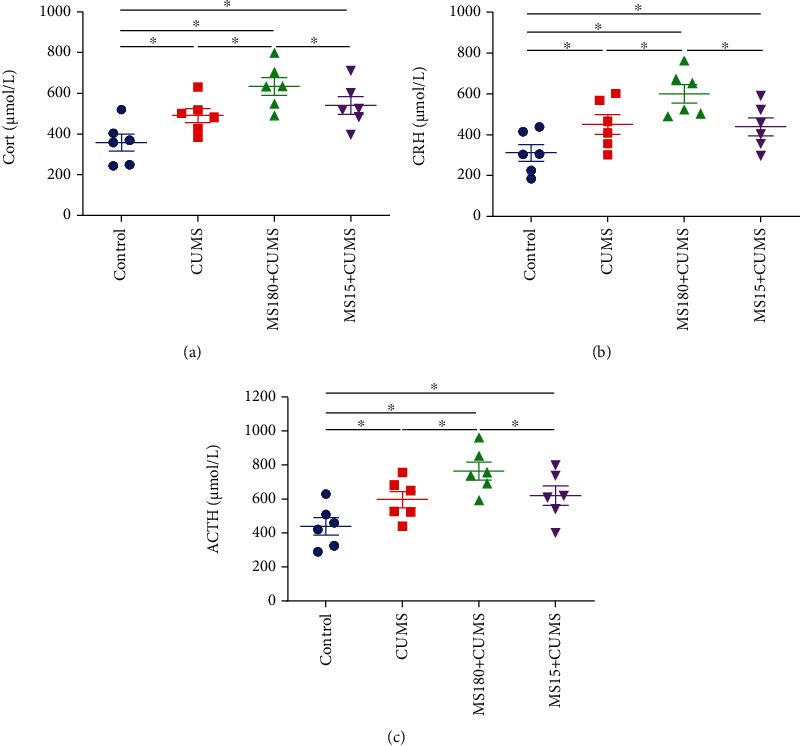
Effects of MS experience on serum hormone levels when exposed to CUMS. (a) The effect of MS on Cort levels when exposed to CUMS. (b) The effect of MS on CRH levels when exposed to CUMS. (c) The effect of MS on the ACTH levels when exposed to CUMS. ^∗^*P* < 0.05 between the two groups.

We found that the levels of Cort, CRH, and ACTH in serum were higher in the CUMS group when compared with the controls. In comparison with the CUMS groups, the levels of Cort (*F* (3, 20) = 7.705, *P* < 0.05; Figure 4(a)), CRH (*F* (3, 20) = 6.905, *P* < 0.05; Figure 4(b)), and ACTH (*F* (3, 20) = 6.420, *P* < 0.05; Figure 4(c)) exhibited a remarkable increase in the MS180+CUMS group. However, there was no significant difference between the MS15+CUMS and CUMS groups (*P* > 0.05). These data indicated that the dysfunction of Cort, CRH, and ACTH induced by prolonged MS might be the reason for the depression-like behavior susceptibility to adult CUMS exposure.

### 3.6. Effects of MS Experience on the Levels of Monoamine Neurotransmitters at PD106 When Exposed to CUMS

**Figure 5:**
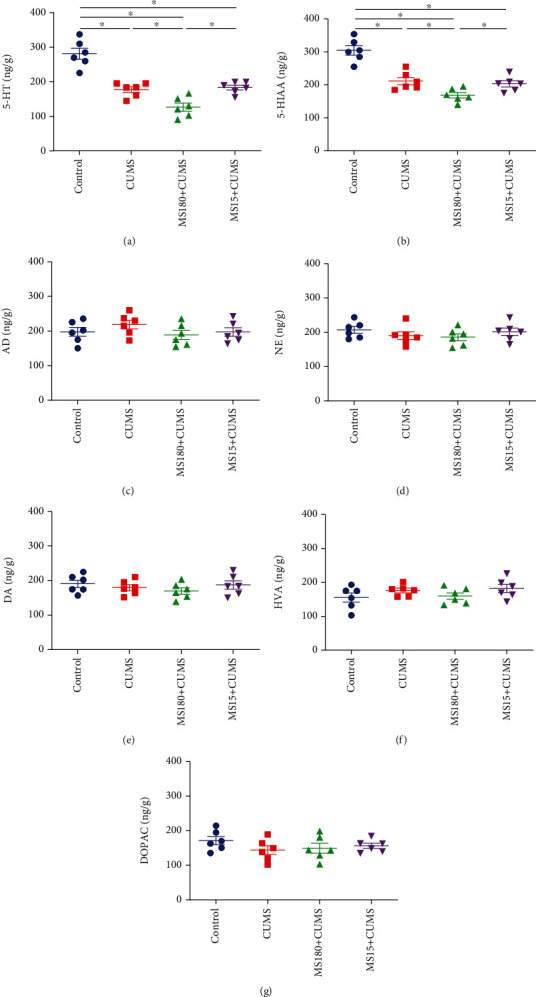
Effects of MS experience on monoamine neurotransmitter levels when exposed to CUMS. (a) The effect of MS on the 5-HT levels when exposed to CUMS. (b) The effect of MS on 5-HIAA levels when exposed to CUMS. (c) The effect of MS on AD levels when exposed to CUMS. (d) The effect of MS on the NE levels when exposed to CUMS. (e) The effect of MS on the DA levels when exposed to CUMS. (f) The effect of MS on HVA levels when exposed to CUMS. (g) The effect of MS on DOPAC levels when exposed to CUMS. ^∗^*P* < 0.05 between the two groups.

When compared with the controls, the monoamine levels of 5-HT and 5-HIAA in the hippocampus were lower in the CUMS group. In comparison with the CUMS group, the levels of 5-HT (*F* (3, 20) = 32.846, *P* < 0.01; Figure 5(a)) and 5-HIAA (*F* (3, 20) = 28.956, *P* < 0.01; Figure 5(b)) showed a significant decrease in the MS180+CUMS group. However, there was no statistical significance between the MS15+CUMS and CUMS groups (*P* > 0.05). Moreover, there was no significant difference of AD (*F* (3, 20) = 1.005, *P* > 0.05; Figure 5(c)), NE (*F* (3, 20) = 0.870, *P* > 0.05; Figure 5(d)), DA (*F* (3, 20) = 0.810, *P* > 0.05; Figure 5(e)), HVA (*F* (3, 20) = 1.429, *P* > 0.05; Figure 5(f)), and DOPAC (*F* (3, 20) = 1.171, *P* > 0.05; Figure 5(g)) within groups. The findings suggested that the alteration of 5-HT and 5-HIAA in the hippocampus induced by prolonged MS might lead to the depression-like behavior susceptibility to CUMS exposure in adulthood.

## 4. Discussion

In the current study, we addressed the critical impacts of early life stress on the behavior response and investigated the alterations of the hippocampal neurosteroids, serum hormones, and hippocampal monoamine neurotransmitters later in life when reexposed to stress. Our results demonstrated that mice in the MS180 group displayed the reduced struggling time and the increased latency to immobility in both FST and TST. The levels of hippocampal neurosteroid (progesterone and allopregnanolone) were decreased and the serum levels of Cort, CRH, and ACTH were overexpressed in the MS180 group. Besides, the expressions of monoamine neurotransmitters such as 5-HT and 5-HIAA significantly decreased in the MS180 group, but not in the MS15 group. All findings revealed that prolonged MS, rather than short MS, could increase the susceptibility to depression-like behavior when reexposed to stress in adulthood.

Short and prolonged MS was used to model early stress adversity as described previously [14]. Separation in early life could exaggerate HPA axis responses to stress in adulthood as well as behavioral and cognitive disturbances [27], which were related to the dysfunction of HPA-associated genes [28]. Some studies reported that MS could exert different functional effects on sensitivity to chronic stress. For example, it reported that MS increasing behavioral stress sensitivity on the response upon social defeat stress in adulthood was through inappropriately preserved brain plasticity in a sex-dependent and region-dependent manner [29]. And the underlying effect was associated with epigenetic changes of H3K4me3 in the murine prefrontal cortex [30]. However, another study reported that MS increased neuroinflammatory activation and inhibited CRMP2-induced neuroprotection, thereby leading to the impairment of neuroplasticity after subsequent exposure to CUMS [31]. To better model depression in humans and detect biochemical changes caused by depression, we used the CUMS model in this study. CUMS was first proposed by Katz et al. [32] and further modified by Willner et al. [33], which is a reliable and effective model for modeling depression in rodents. Although both MS and CUMS models have been well studied in depression, there are conflicting reports on the degree of early life adversity on stress reexposure in adulthood. Herein, maternally separated mice that were exposed at different time points were subjected to a series of unexpected stressors over a few weeks, and certain behavior performance and neural variables were investigated.

In the FST and TST, the immobility time was measured to learn helplessness in response to despair. In this study, we found that the struggling time was decreased and the immobility time was increased in FST and TST in the MS180 group. However, there was no significant difference in the MS15 group. It indicated that the changes in adult depression-like behaviors were induced by the prolonged MS experience. The results were consistent with the previous studies [31, 34, 35]. About OFT, it is applied to measure spontaneous activity. The path length in the OFT is also named total distance traversed which is used to exclude the potential factors other than psychiatric disorders induced by stress adversity. It showed that there were no significant changes among groups. All data demonstrated that long-term separation could promote behavioral susceptibility of mice to adult stress reexposure, while short-term separation did not develop depression-like behaviors. The findings are in line with a published study that prolonged MS may be susceptible to generate mental disorders later in life [20].

The pathogenesis of depression involving the HPA axis, neuroendocrine disorders, and monoamine neurotransmitters is the most widely studied and recognized hypotheses. Among them, the HPA axis is the main focus of stress response [36]. Long-term exposure to stress leads to the overproduction of Cort, CRH, and ACTH, which affects the activity of glucocorticoid receptors, thereby disturbing the negative feedback of HPA axis function and causing HPA axis hyperactivity [37]. In our study, we found that the serum levels of Cort, CRH, and ACTH are overexpressed after prolonged MS exposure. However, no significant difference was found in the short-term MS. The results are consistent with a previous study [38]. Neuroactive steroid progesterone and allopregnanolone are stress-responsive, stress-reducing hormones, which have pivotal effects on stress physiology. Multiple evidences implicated that the expressions of progesterone and allopregnanolone were decreased in depressive disorders [39, 40]. And other studies implicated that the increased levels of allopregnanolone could have antidepressant effects [41, 42]. In our experiment, we found that the progesterone and allopregnanolone levels were significantly decreased in prolonged early stress, suggesting that progesterone and allopregnanolone may act as potential contributors to depression. Neurotransmitters that carry messages play crucial roles in the regulation of neuroplasticity. The imbalance of monoaminergic neurotransmitters is intimately involved in the occurrence and development of stress-induced depression [43]. We found that 5-HT and 5-HIAA were significantly decreased in long-term MS. However, there was no significant difference in short-term MS. 5-HT deficiency has been proven to be closely related to the increased vulnerability to depression [44]. Our findings were also supported by a previous study [45]. It said that the levels of 5-HT and 5-HIAA were downregulated after prolonged stress adversity.

The major limitation of our study is that we only focus on the altered depression-like phenotypes in the hippocampus. Other regions such as the prefrontal cortex, amygdala, and nucleus accumbens are also important locus of depression [41, 42]. Future studies involving different regions to explore the underlying susceptible pathophysiology of depression are needed.

In conclusion, the present study confirmed the different effects of childhood stress adversity on the susceptibility to depression-like behavior when reexposed to stress in adulthood. We highlighted that prolonged MS could induce stress vulnerability, which might be related to the alteration of the hippocampal neurosteroids, serum hormones, and hippocampal monoamine neurotransmitters. However, researches on the underlying neuromolecular mechanism of the early life experience on the depression-like behavior susceptibility to stress reexposure later in life are needed in the future.

## Figures and Tables

**Table 1:** Representative schedule^a^ of stressors used during the one-week intervention.

Day of intervention	Stressor used	Duration
1	Wet sawdust	24 h
2	Water deprivation	24 h
3	No stressor applied	—
4	Flashing light (stroboscopic light, 60 flashes/min)	3 h
5	Restraint (80 cm^3^ breathable, transparent, and plastic pipe)	6 h
6	Food deprivation	24 h
7	Foot shock (electric foot shocks, intensity: 1 mA, duration: 4 s, interval: 26 s)	20 min

^a^The weekly schedule was randomly generated based on the above seven stressors.

**Table 2:** Results of repeated measures ANOVA of MS experience on FST, TST, and OFT levels when exposed to CUMS.

Time point	Group vs. group	FST	TST	OFT
PD63	Control *vs.* CUMS	*t* = −0.095, *P* = 0.926	*t* = −0.687, *P* = 0.507	*t* = −0.880, *P* = 0.400
	Control *vs.* MS180+CUMS	*t* = −0.363, *P* = 0.724	*t* = −0.445, *P* = 0.666	*t* = 0.299, *P* = 0.771
	Control *vs.* MS15+CUMS	*t* = −0.207, *P* = 0.840	*t* = −0.126, *P* = 0.903	*t* = 0.137, *P* = 0.894
	CUMS *vs.* MS180+CUMS	*t* = −0.312, *P* = 0.761	*t* = 0.239, *P* = 0.816	*t* = −0.582, *P* = 0.574
	CUMS *vs.* MS15+CUMS	*t* = −0.139, *P* = 0.892	*t* = 0.541, *P* = 0.601	*t* = −0.6861, *P* = 0.508
	MS180+CUMS *vs.* MS15+CUMS	*t* = 0.133, *P* = 0.897	*t* = 0.308, *P* = 0.764	*t* = −0.140, *P* = 0.892
PD70	Control *vs.* CUMS	*t* = −0.586, *P* = 0.571	*t* = −1.211, *P* = 0.254	*t* = 0.644, *P* = 0.534
	Control *vs.* MS180+CUMS	*t* = −0.953, *P* = 0.363	*t* = −3.073, *P* = 0.012^∗^	*t* = 0.292, *P* = 0.777
	Control *vs.* MS15+CUMS	*t* = −0.292, *P* = 0.776	*t* = −1.057, *P* = 0.315	*t* = −0.331, *P* = 0.747
	CUMS *vs.* MS180+CUMS	*t* = −0.236, *P* = 0.819	*t* = −1.318, *P* = 0.217	*t* = −0.398, *P* = 0.699
	CUMS *vs.* MS15+CUMS	*t* = 0.225, *P* = 0.827	*t* = −0.169, *P* = 0.869	*t* = −1.068, *P* = 0.311
	MS180+CUMS *vs.* MS15+CUMS	*t* = 0.470, *P* = 0.649	*t* = 0.831, *P* = 0.425	*t* = −0.684, *P* = 0.509
PD77	Control *vs.* CUMS	*t* = −0.588, *P* = 0.570	*t* = −2.480, *P* = 0.033^∗^	*t* = 0.061, *P* = 0.952
	Control *vs.* MS180+CUMS	*t* = −1.362, *P* = 0.203	*t* = −3.101, *P* = 0.011^∗^	*t* = 0.205, *P* = 0.842
	Control *vs.* MS15+CUMS	*t* = −1.081, *P* = 0.305	*t* = −1.950, *P* = 0.080	*t* = −0.467, *P* = 0.650
	CUMS *vs.* MS180+CUMS	*t* = −0.963, *P* = 0.358	*t* = −1.625, *P* = 0.135	*t* = 0.119, *P* = 0.908
	CUMS *vs.* MS15+CUMS	*t* = −0.482, *P* = 0.640	*t* = 0.008, *P* = 0.994	*t* = −0.460, *P* = 0.656
	MS180+CUMS *vs.* MS15+CUMS	*t* = 0.611, *P* = 0.555	*t* = 1.460, *P* = 0.175	*t* = −0.657, *P* = 0.526
PD84	Control *vs.* CUMS	*t* = −2.330, *P* = 0.042^∗^	*t* = −4.871, *P* = 0.001^∗^	*t* = −0.283, *P* = 0.783
	Control *vs.* MS180+CUMS	*t* = −3.399, *P* = 0.007^∗^	*t* = −5.245, *P* < 0.001^∗^	*t* = −0.180, *P* = 0.861
	Control *vs.* MS15+CUMS	*t* = −2.133, *P* = 0.059	*t* = −3.510, *P* = 0.006^∗^	*t* = 0.380, *P* = 0.712
	CUMS *vs.* MS180+CUMS	*t* = −2.231, *P* = 0.050	*t* = −1.450, *P* = 0.178	*t* = 0.080, *P* = 0.938
	CUMS *vs.* MS15+CUMS	*t* = −0.130, *P* = 0.899	*t* = 0.592, *P* = 0.567	*t* = 0.638, *P* = 0.538
	MS180+CUMS *vs.* MS15+CUMS	*t* = 2.072, *P* = 0.065	*t* = 1.824, *P* = 0.098	*t* = 0.512, *P* = 0.620
PD91	Control *vs.* CUMS	*t* = −2.898, *P* = 0.016^∗^	*t* = −4.813, *P* = 0.001^∗^	*t* = −0.474, *P* = 0.646
	Control *vs.* MS180+CUMS	*t* = −5.487, *P* = 0.001^∗^	*t* = −6.388, *P* < 0.001^∗^	*t* = 0.126, *P* = 0.902
	Control *vs.* MS15+CUMS	*t* = −3.101, *P* = 0.011^∗^	*t* = −3.947, *P* = 0.003^∗^	*t* = −0.386, *P* = 0.708
	CUMS *vs.* MS180+CUMS	*t* = −2.196, *P* = 0.053	*t* = −2.084, *P* = 0.064	*t* = −0.589, *P* = 0.569
	CUMS *vs.* MS15+CUMS	*t* = 0.176, *P* = 0.864	*t* = 0.589, *P* = 0.569	*t* = 0.127, *P* = 0.902
	MS180+CUMS *vs.* MS15+CUMS	*t* = 2.530, *P* = 0.030^∗^	*t* = 2.571, *P* = 0.028^∗^	*t* = −0.512, *P* = 0.619
PD98	Control *vs.* CUMS	*t* = −3.081, *P* = 0.012^∗^	*t* = 4.507, *P* = 0.001^∗^	*t* = −0.390, *P* = 0.705
	Control *vs.* MS180+CUMS	*t* = −5.940, *P* < 0.001^∗^	*t* = −7.161, *P* < 0.001^∗^	*t* = 0.174, *P* = 0.866
	Control *vs.* MS15+CUMS	*t* = −3.066, *P* = 0.012^∗^	*t* = −5.018, *P* = 0.001^∗^	*t* = 1.378, *P* = 0.198
	CUMS *vs.* MS180+CUMS	*t* = −2.281, *P* = 0.046^∗^	*t* = −2.672, *P* = 0.023^∗^	*t* = 0.568, *P* = 0.583
	CUMS *vs.* MS15+CUMS	*t* = −0.108, *P* = 0.916	*t* = 0.642, *P* = 0.642	*t* = 1.695, *P* = 0.121
	MS180+CUMS *vs.* MS15+CUMS	*t* = 2.108, *P* = 0.061	*t* = 2.203, *P* = 0.052	*t* = 1.284, *P* = 0.228
PD105	Control *vs.* CUMS	*t* = −3.013, *P* = 0.013^∗^	*t* = −4.093, *P* = 0.002^∗^	*t* = 0.429, *P* = 0.677
	Control *vs.* MS180+CUMS	*t* = −6.167, *P* < 0.001^∗^	*t* = −7.223, *P* < 0.001^∗^	*t* = 0.651, *P* = 0.530
	Control *vs.* MS15+CUMS	*t* = −3.325, *P* = 0.008^∗^	*t* = −4.524, *P* = 0.001^∗^	*t* = 0.241, *P* = 0.814
	CUMS *vs.* MS180+CUMS	*t* = −3.188, *P* = 0.010^∗^	*t* = −2.777, *P* = 0.020^∗^	*t* = 0.210, *P* = 0.838
	CUMS *vs.* MS15+CUMS	*t* = −0.283, *P* = 0.783	*t* = 0.107, *P* = 0.917	*t* = −0.108, *P* = 0.916
	MS180+CUMS *vs.* MS15+CUMS	*t* = 2.923, *P* = 0.015^∗^	*t* = 3.080, *P* = 0.012^∗^	*t* = −0.281, *P* = 0.785

^∗^ *P* < 0.05 between the two groups.

## Data Availability

The data used to support the findings of this study are included in the article.
